# Dermoscopy of Melanomas on the Trunk and Extremities in Asians

**DOI:** 10.1371/journal.pone.0158374

**Published:** 2016-07-08

**Authors:** Je-Ho Mun, Jungyoon Ohn, Woo-Il Kim, Sung-Min Park, Moon-Bum Kim

**Affiliations:** 1 Department of Dermatology, Seoul National University School of Medicine, Seoul, Korea; 2 Institute of Human-Environment Interface Biology, Seoul National University, Seoul, Korea; 3 Department of Dermatology, Pusan National University College of Medicine, Busan, Korea; 4 Medical Research Institute, Pusan National University Hospital, Busan, Korea; University of Queensland Diamantina Institute, AUSTRALIA

## Abstract

The incidence of melanoma among the Asian population is lower compared to that among the Western European population. These populations differed in their most common histopathologic subtypes, acral lentiginous melanoma being the most common in the Asian population. Although the dermoscopic features of the melanomas on the acral skin have been thoroughly investigated in the Asian population, studies concerning the dermoscopic patterns of melanomas on the non-acral skin have been scarce. The aim of this study was to investigate the dermoscopic patterns of melanomas on the trunk and extremities in the Asian population. To achieve this, we evaluated the dermoscopic patterns of 22 primary melanomas diagnosed at two university hospitals in Korea. In addition, 100 benign melanocytic lesions were included as the control group for comparative analysis. A P value less than 0.05 was regarded as statistically significant. Melanoma-associated dermoscopic features such as asymmetry (odds ratio [OR], 30.00), multicolor pattern (OR, 30.12), blotches (OR, 13.50), blue white veils (OR, 15.75), atypical pigment networks (OR, 9.71), irregular peripheral streaks (OR, 6.30), atypical vascular patterns (OR, 11.50), ulcers (OR, 15.83), atypical dots/globules (OR, 3.15), shiny white lines (OR, 5.88), and regression structures (OR, 7.06) were more commonly observed in patients with melanomas than in patients of the control group. The mean dermoscopic scores obtained on the 7-point checklist, revised 7-point checklist, 3-point checklist, ABCD rule, and CASH algorithm were 5.36, 3.41, 2.05, 6.89, and 9.68, respectively, in the primary melanomas, and 1.33, 0.93, 0.46, 2.45, and 3.60, respectively, in the control group (all, P < 0.001). The present study showed that melanoma-related dermoscopic patterns were common in Asian patients. Dermoscopy is a reliable diagnostic tool for the melanomas of the trunk and extremities in the Asian populations.

## Introduction

Malignant melanoma (MM) is among the most aggressive and treatment-resistant human cancers [1]. It has been an increasingly important public health problem worldwide [2]. The incidence of melanoma has been steadily increasing 4–6% annually in the USA [3]. Although the incidence of melanoma in the Asian population is lower than that in the Western European population, melanoma is the most common cause of cancer-related mortality among Asian patients with skin cancers [4]. Because of the low incidence rates and low public awareness associated with MM, its diagnosis is often delayed, resulting in more advanced stages of disease at presentation among the individuals of non-European descent [5]. The early detection of melanoma is crucial for a favorable prognosis because prognosis is directly associated with the invasion depth of the melanoma.

Dermoscopy improves the diagnostic accuracy rates for melanomas by 49% compared to the clinical diagnosis with the naked eye [6]. Although the dermoscopic patterns of melanomas have been widely reported in the Western European population, data on the dermoscopic patterns of the melanoma of the trunk in the Asian population are scarce. This is attributed to the fact that the most common histological subtype is acral lentiginous melanoma, constituting roughly 45–66% of the cases compared to the 2–3% of all cases in patients from Western European descent [7–9]. Therefore, previous dermoscopic studies on the melanomas in the Asian population were mainly focused on the acral melanomas [10–12]. Because dermoscopic patterns of the melanomas on the non-glabrous skin are different from acral melanomas, a study investigating the dermoscopic features of the melanomas on the trunk in the Asian population is necessary. Although the dermoscopic principles and the fundamental patterns of melanomas are the same regardless of the race, the differences between the skin and demographic characteristics of the Asian patients and those from the Western European descent necessitated this study.

## Materials and Methods

The patients who were diagnosed with MM at the Seoul National University Hospital, Seoul, and Pusan National University Hospital, Busan, between 2007 and 2014 were enrolled. The inclusion criteria were as follows: 1) histopathologically confirmed melanomas, 2) anatomic location of the trunk and limb with the acral, facial, and other special areas excluded, 3) availability of high quality clinical and dermoscopic photography. Dermoscopic photographs were acquired using a dermatoscope (DermLite II Pro HR or DL3 equipment) attached to a digital camera. The images were acquired under cross-polarized light without any liquid medium. For the comparative analysis, we included benign nevi on the trunk and the extremities (arm and leg) for the control group.

We evaluated the images according to the melanoma-related patterns that were previously described in the literature [13–16]. In addition, we calculated the scores for each lesion using dermoscopic algorithms such as the 7-point checklist, revised 7-point checklist, 3-point checklist, ABCD rule, and the color, architecture, symmetry, and homogeneity (CASH) algorithm [17–21]. The evaluation was performed by two dermatologists experienced in dermoscopy without knowledge of the final diagnosis (JHM and WIK). All statistical analyses were performed using the IBM SPSS version 21. We used simple cross-tabulations and the Pearson chi-square or Fisher exact test for the comparison of the proportions. Student t-test was used to analyze the continuous variables. Odds ratios (ORs) with the corresponding 95% confidence intervals (CIs) were calculated. Inter-observer agreement was examined using the Cohen kappa. All given *p* values were 2-tailed, and *p* values ≤0.05 (95% confidence) were considered statistically significant.

The present study was approved by the institutional review board of the Seoul National University Hospital (IRB No.: H-1510-120-714), and it was conducted in compliance with the principles of the Declaration of Helsinki. The need for informed consent for participation in this study was waived because the data was analyzed anonymously. The clinical and dermoscopic photographs were obtained with the patients’ consent during skin examination, and they agreed that the images could be used in research if their identity would not be disclosed.

## Results

During the study period, 276 patients were diagnosed with MM. MM occurred on the acral area (n = 180, 65.2%), head and neck (n = 31, 11.2%), and the trunk and the extremities (n = 65, 23.6%). Among these cases, only those that were anatomically located in the trunk and the limbs were enrolled; the acral, facial, and other special areas were excluded. In addition, we excluded patients with unavailable or poor clinical and dermoscopic photographs. Finally, 22 MM cases were analyzed in the present study. The mean age of the patients with MM was 59.4 years (range, 32–84 years), and 63.6% (n = 14) of the patients were women. Nineteen cases were invasive melanomas (86.4%), and three cases were melanoma *in situ*. Twelve cases were superficial spreading melanomas, and 10 cases were nodular type. The mean Breslow’s thickness was 3.4 ± 4.0 mm.

The control group consisted of 100 benign nevi including 86 banal melanocytic nevi, 6 congenital nevi, 5 intradermal nevi, 2 Spitz nevi, and 1 blue nevus. Thirty-seven cases were histopathologically examined. We included 63 cases of non-biopsied banal nevi to avoid a selection bias because only difficult or suspicious nevi were excised, and majority of the typical lesions were managed with observation. The diagnosis of banal nevi was made based on typical clinical and dermoscopic findings.

In primary melanomas, the following melanoma-associated dermoscopic patterns were detected: asymmetry (90.9%), blotches (81.8%), multicolor patterns (81.8%), blue-white veils (63.6%), atypical pigment networks (52.5%), irregular peripheral streaks (54.5%), atypical vascular patterns (50.0%), ulcers (45.5%), atypical dots/globules (40.9%), white shiny lines (27.3%), brown peripheral structureless areas (22.7%), and regression structures (22.7%). The comparison of the dermoscopic features between the primary melanoma group and the control group is demonstrated in Table 1.

**Table 1:** The frequency distributions of dermoscopic patterns between melanomas and benign nevi showing significant associations.

Dermoscopic patterns	Melanoma (N = 22), n (%)	Benign nevus (N = 100), n (%)	OR	95% CI	P value
Asymmetry	20 (90.9)	25 (25.0)	30.00	6.55–137.50	<0.001
Multicolor pattern	18 (81.8)	13 (13.0)	30.12	8.80–103.05	<0.001
Irregular blotches	18 (81.8)	25 (25.0)	13.50	4.17–43.68	<0.001
Blue-white veil	14 (63.6)	10 (10.0)	15.75	5.31–46.70	<0.001
Atypical pigment network	12 (54.5)	11 (11.0)	9.71	3.41–27.67	<0.001
Irregular peripheral streaks	12 (54.5)	16 (16.0)	6.30	2.33–17.04	<0.001
Atypical vascular patterns	11 (50.0)	8 (8.0)	11.50	3.81–34.71	<0.001
Ulcers	10 (45.5)	5 (5.0)	15.83	4.63–54.17	<0.001
Atypical dots/globules	9 (40.9)	18 (18.0)	3.15	1.17–8.50	0. 026
Shiny white lines	6 (27.3)	6 (6.0)	5.88	1.68–20.50	0.008
Brown peripheral structureless area	5 (22.7)	12 (12.0)	2.15	0.67–6.72	0.189
Regression structure	5 (22.7)	4 (4.0)	7.06	1.72–28.98	0.010

**Fig 1:**
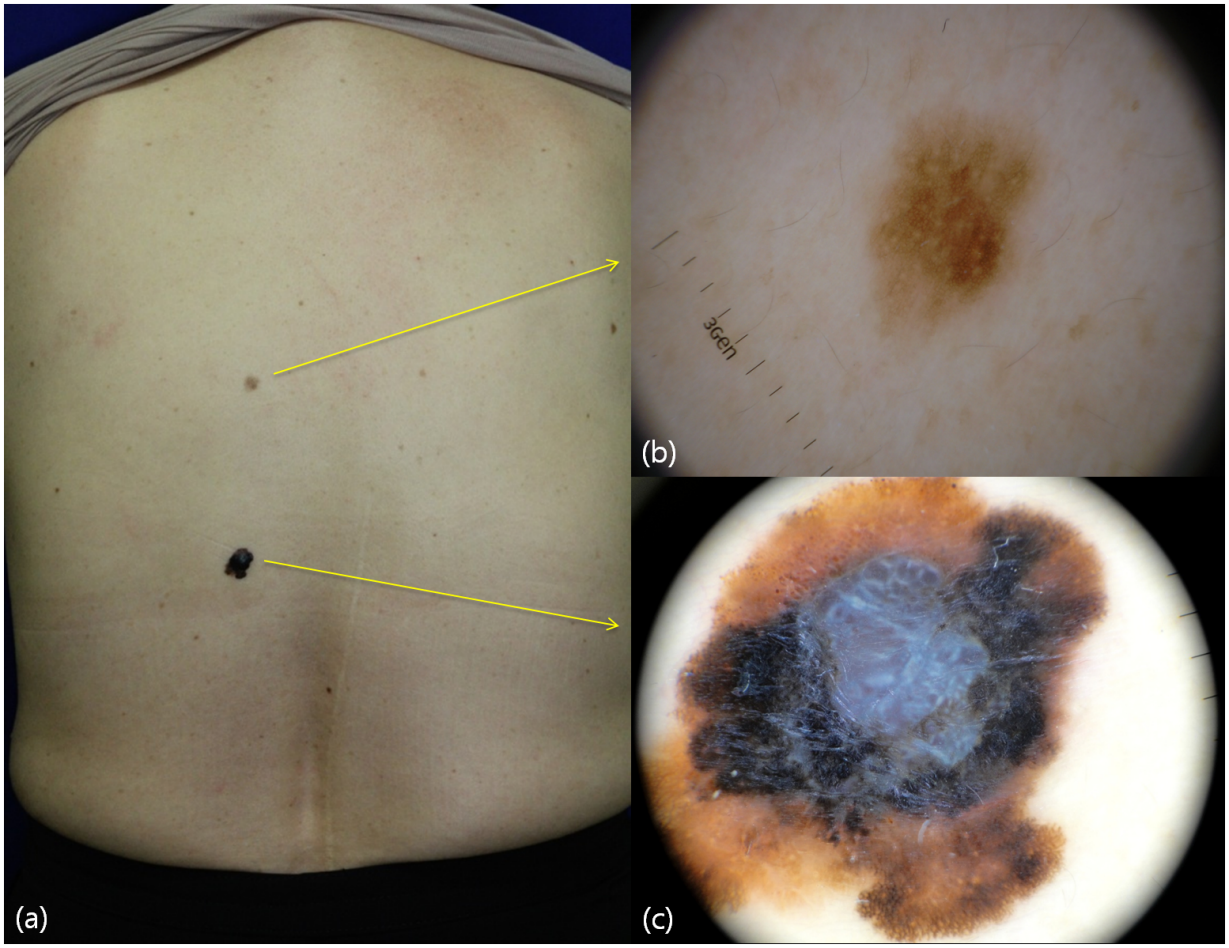
Clinical image and dermoscopic features of a malignant melanoma and benign nevus in a 60-year-old woman. Compared to the clinical picture (a), dermoscopic evaluation provides detailed clues for the diagnosis of melanomas (b), dermoscopy of benign nevus showing a reticular pattern (c). Dermoscopy of malignant melanoma exhibits melanoma-associated patterns such as asymmetry, atypical pigment networks, blue-white veil, irregular blotches, irregular dots, peripheral streaks, shiny white lines, and multicolor patterns.

**Fig 2:**
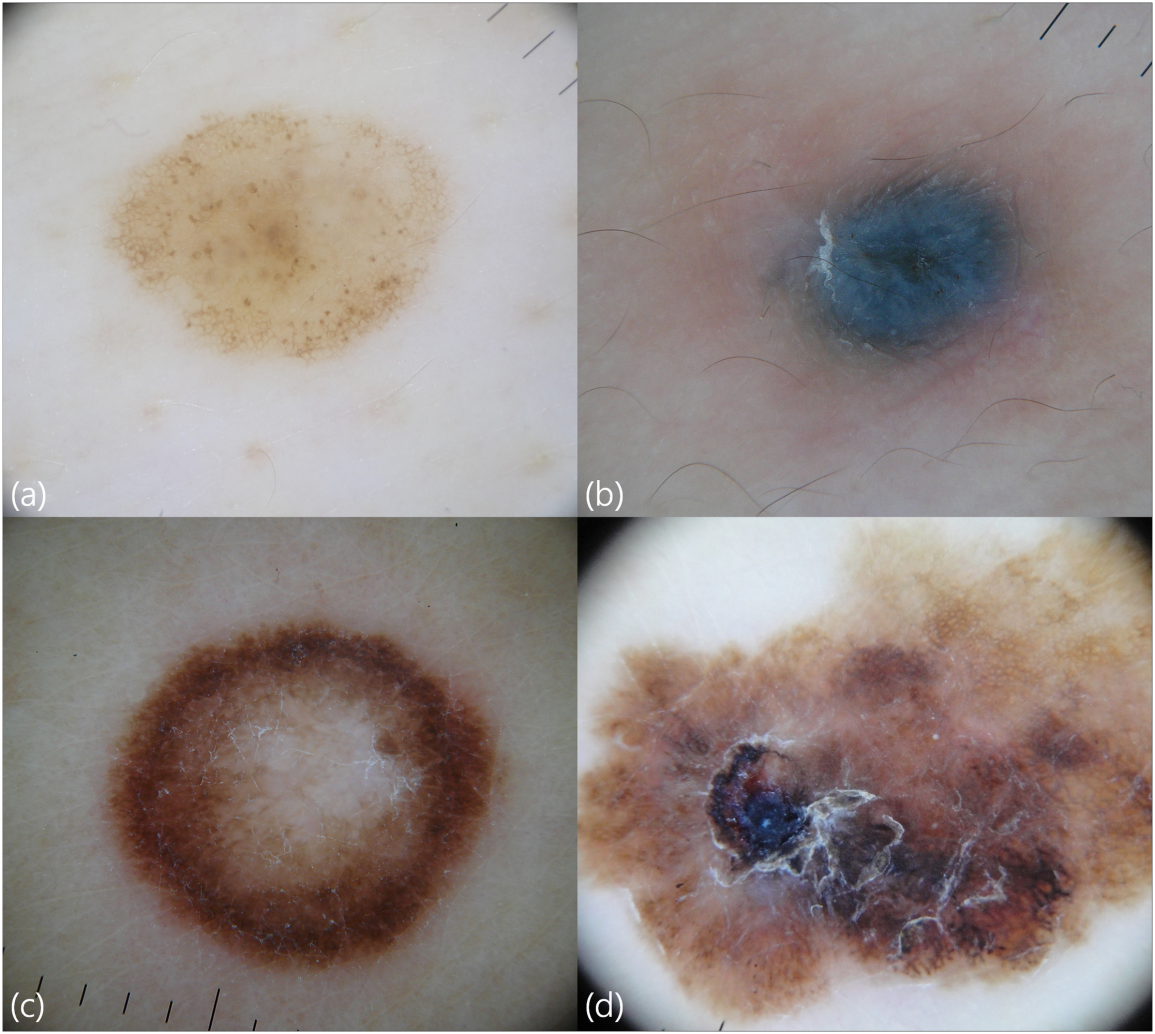
Dermoscopic features of a melanocytic nevus with a globular and reticular pattern (a), a blue nevus (b), a spitz nevus (c), and a malignant melanoma (d). Compared to benign nevi, a melanoma exhibits more patterns such as asymmetry, irregular blotches, atypical pigment networks, irregular dots, peripheral streaks, multicolor patterns, and ulcer.

In melanomas, the more commonly observed dermoscopic patterns were the following: asymmetry (OR, 30.00; 95% CI, 6.55–137.50; P < 0.001); multicolor pattern (OR, 30.12; 95% CI, 8.80–103.05; P < 0.001); blotches (OR, 13.50; 95% CI, 4.17–43.68; P < 0.001); blue white veils (OR, 15.75; 95% CI, 5.31–46.70; P < 0.001); atypical pigment networks (OR, 9.71; 95% CI, 3.41–27.67; P < 0.001); irregular peripheral streaks (OR, 6.30; 95% CI, 2.33–17.04; P < 0.001); atypical vascular patterns (OR, 11.50; 95% CI, 3.81–34.71.76; P < 0.001); ulcers (OR, 15.83; 95% CI, 4.63–54.17; P < 0.001); atypical dots/globules (OR, 3.15; 95% CI, 1.17–8.50; P = 0.026); shiny white lines (OR, 5.88; 95% CI, 1.68–20.50; P = 0.008); and regression structures (OR, 7.06; 95% CI, 1.72–28.98; P = 0.010). The brown peripheral structureless areas did not differ significantly from the benign lesions (OR, 2.15; 95% CI, 0.67–6.72; P = 0.189) (Figs 1 and 2).

The mean scores on the 7-point checklist, revised 7-point checklist, 3-point checklist, ABCD rule, and CASH algorithm were 5.36 ± 1.79, 3.41 ± 1.33, 2.05 ± 0.90, 6.89 ± 2.10, and 9.68 ± 2.57 in primary melanomas, and 1.33 ± 1.72, 0.93 ± 1.13, 0.46 ± 0.76, 2.45 ± 2.03, and 3.60 ± 2.54 in the control group, respectively. The score differences between the primary melanomas and the control group were statistically significant for all algorithms (all, P < 0.001). The sensitivity, specificity, positive predictive value, and negative predictive value for each algorithm were as follows: 95.5%, 79.0%, 50.0%, 98.8% for the 7-point checklist; 100%, 48.0%, 29.7%, 100% for the revised 7-point checklist; 90.9%, 68.0%, 38.5%, 97.1% for the 3-point checklist; 81.8%, 83.0%, 51.4%, 95.4% for the ABCD rule; and 81.8%, 88.0%, 60.0%, 95.7% for the CASH algorithm.

Cohen kappa ranged from 0.554 to 0.804, showing moderate to excellent agreement between the 2 observers for all variables (asymmetry, multicolor pattern, blotches, blue-white veils, atypical pigment networks, irregular peripheral streaks, atypical vascular patterns, ulcers, atypical dots/globules, shiny white lines, brown peripheral structureless areas, and regression structures).

## Discussion

Dermoscopy is a rapid, noninvasive magnifying tool that allows clinicians to visualize morphologic structures that are not discernible to the naked eye [22]. It improves diagnostic sensitivity for melanomas (90%) compared to that achieved with the naked eye (74%) [23]. Although data on the dermoscopic patterns of melanomas have been widely reported in the Western European population, the data on the dermoscopic patterns of malignant melanomas on the trunk have been scarce in the Asian population. To our knowledge, this is the first original study to report the dermoscopic features of malignant melanomas on the trunk and the limbs, excluding the acral skin, in the Asian population. In melanomas, the commonly observed dermoscopic patterns were asymmetry, blotches, multicolor patterns, blue-white veils, atypical pigment networks, irregular peripheral streaks, atypical vascular patterns, ulcers, atypical dots/globules, white shiny lines, brown peripheral structureless areas, and regression structures.

Asymmetry was determined by visually dividing the lesion into two perpendicular axes to evaluate them in terms of structures or colors. Asymmetry in 2 axes was described as a criterion found in 94–96% of MMs [24]. In our study, asymmetry was the most common finding among the primary melanomas (90.9%). Melanomas frequently show more colors, while benign nevi usually have only one or two colors. Therefore, the assessment of the number of colors (light brown, dark brown, gray, black, red, and white) in a lesion could help discriminate between the benign nevi from melanomas. In this study, multicolor patterns (more than four colors) were more commonly observed (81.8%). Invasion might be associated with an increasing variety of colors [25]. The high detection rate of a multicolor pattern could be attributed to the fact that the majority of the cases were invasive melanomas.

Because the melanocytes in melanomas grow haphazardly in the vertical and horizontal planes, the pigmentary structures are common. In the present study, melanomas showed irregular blotches, blue-white veils, atypical pigment networks, irregular peripheral streaks, atypical dots/globules, and brown structureless areas in 81.8% 63.6%, 54.5%, 54.5%, 40.9%, and 22.7% of the primary melanomas, respectively. All structures showed statistical significance except for the brown structureless areas. This could be attributed to the fact that the majority of cases in the present study were invasive melanomas as brown structureless areas are usually found in thin melanomas [26].

Atypical vascular structures in melanomas are associated with tumor-induced angiogenesis [16]. Therefore, the presence of vessels increases the possibility of melanoma development. In the present study, 50% of the melanomas had atypical vascular structures. The presence of shiny white lines, also called shiny white streaks or chrysalis structures, increased the possibility of malignant tumors [27, 28]. In this study, shiny white lines were observed in 27.3% of the melanomas. Regression structures are white scar-like areas with peppering overlying flat or thin portions of a lesion [16]. They were found in 22.7% of the melanomas. An ulcer is a non-specific finding. However, it can increase the index of a malignant tumor, especially when it occurs without apparent trauma. In our data, only 5% of benign nevi showed ulcers, while 45.5% of melanomas had ulcers.

Different dermoscopic algorithmic methods for melanocytic lesions were developed to discriminate melanomas from benign nevi. In the present study, the mean scores of the 7-point checklist, revised 7-point checklist, 3-point checklist, ABCD rule, and the CASH algorithm in melanomas were 5.36, 3.41, 2.05, 6.89, and 9.68, respectively. These algorithms had 81.8–100% sensitivity in discriminating melanomas from benign nevi with modest to high specificities (48–88%). Recently, Argenziano et al. revised their 7-point checklist [29]. They lowered the threshold to increase the sensitivity for optimizing melanoma screening, which warranted excision only if one of the seven criteria was observed [29]. In the present study, the sensitivity of the 7-point checklist was the highest (100%); however, its specificity was the lowest (48.0%).

There were some limitations to the present study. First, only a small number of melanomas were included and the majority of the cases were in advanced stages. Therefore, further studies with larger samples and the inclusion of thin melanomas in the Asian population are necessary. Second, we did not include data from the non-Asian group. Therefore, we could not compare the dermoscopic characteristics of the melanoma in the Asian and the Western European groups. Future studies should be conducted to compare the features of the melanomas.

In conclusion, we showed that melanoma-related dermoscopic patterns are common among Asian patients. Consequently, dermoscopy is a reliable tool in diagnosing melanomas of the trunk and the extremities in the Asian population. Compared to Western European population, the use of dermoscopy in Asia is not widespread, and melanomas are diagnosed at rather advanced stages. In this regard, the daily use of dermoscopy might facilitate the detection of melanomas at an early stage. We hope that our report will encourage the popularization of dermoscopy in Asian countries.
