# Correction: Dermoscopy of Melanomas on the Trunk and Extremities in Asians

**DOI:** 10.1371/journal.pone.0161419

**Published:** 2016-08-15

**Authors:** Je-Ho Mun, Jungyoon Ohn, Woo-Il Kim, Sung-Min Park, Moon-Bum Kim

There are errors in affiliation 1 for authors Je-Ho Mun and Jungyoon Ohn. There are errors in affiliation 3 for authors Woo-Il Kim, Sung-Min Park, and Moon-Bum Kim. The correct affiliations are: 1. Department of Dermatology, Seoul National University College of Medicine, Seoul, Korea, and 3. Department of Dermatology, Pusan National University School of Medicine, Busan, Korea.
